# Supplementary material for: Associations between Temperature and Influenza Activity: A National Time Series Study in China
Source: Int J Environ Res Public Health. 2021 Oct 15;18(20):10846. doi: 10.3390/ijerph182010846 (PMC8535740; doi:10.3390/ijerph182010846)
Supplement: Supplementary file 1 [file ijerph-18-10846-s001.zip › ijerph-1388526-supplementary.pdf]

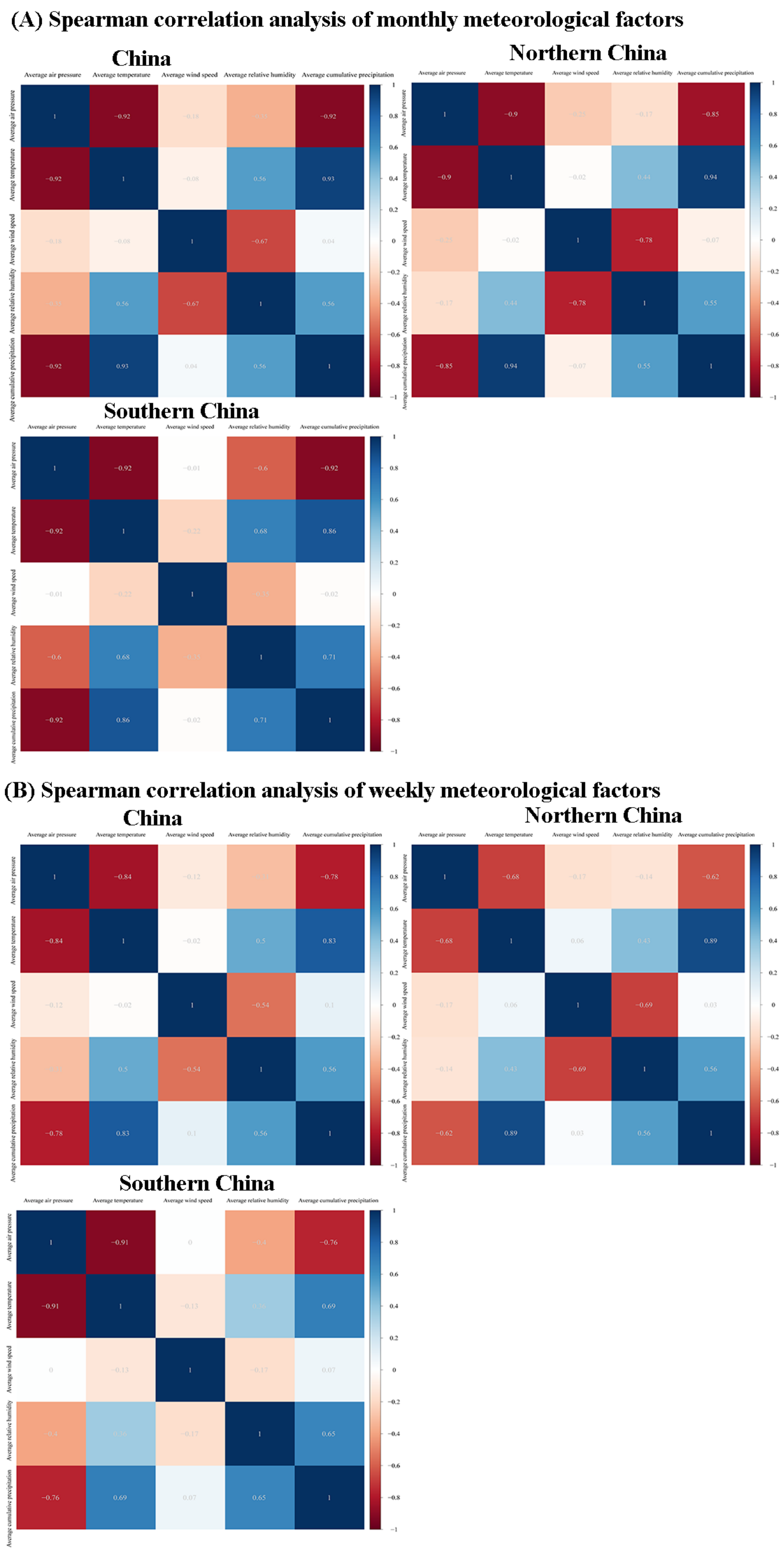

Supplementary Figure S1. The Spearman correlation analysis of meteorological factors

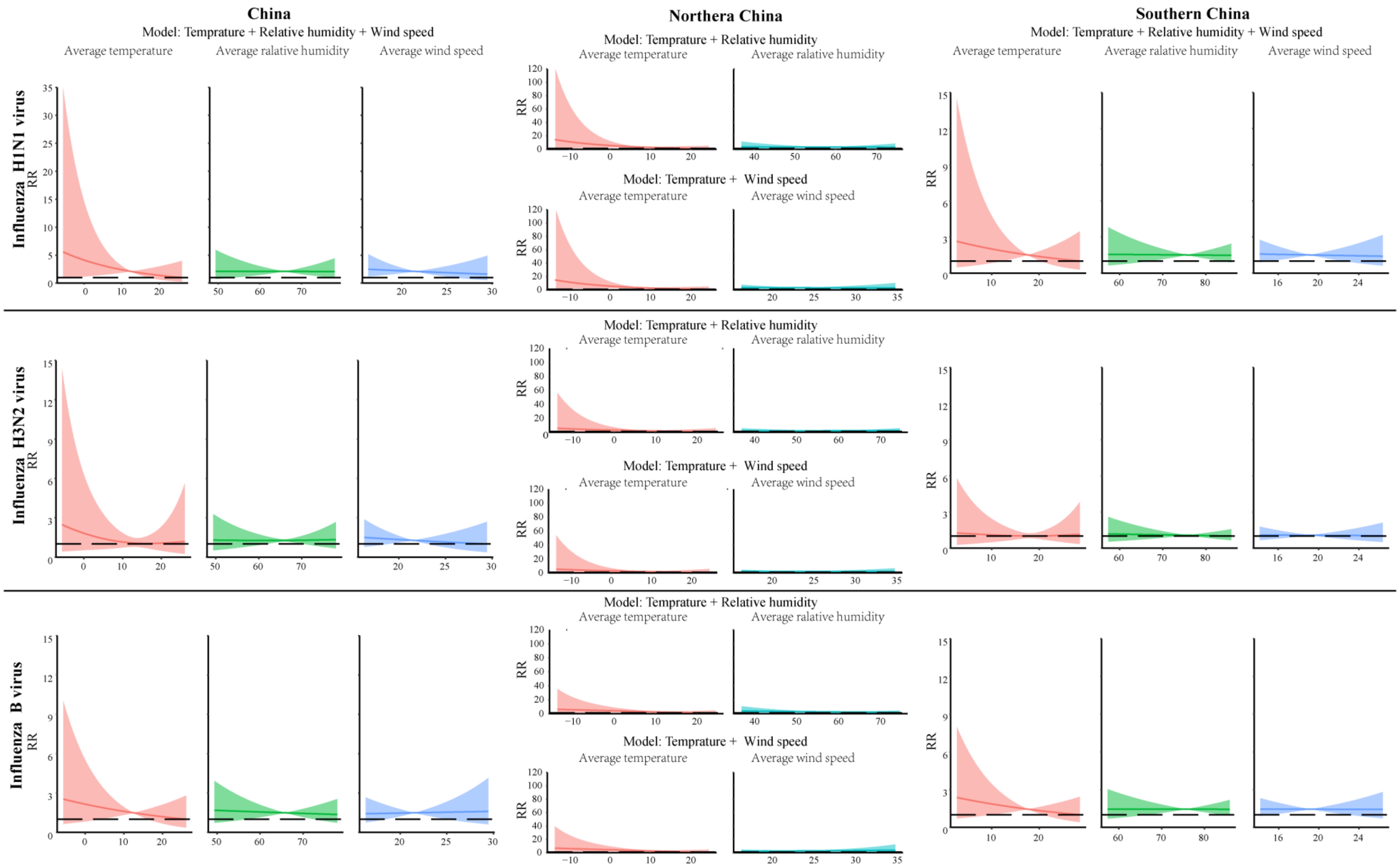

Supplementary Figure S2. The exposure-response of weekly AT to the positive rate of influenza virus
